# Patient Profiles in the Utilization of the CanGaroo® Envelope

**DOI:** 10.7759/cureus.12702

**Published:** 2021-01-14

**Authors:** Hemal Nayak, Andrew D Beaser, Zaid A Aziz

**Affiliations:** 1 Cardiac Electrophysiology, University of Chicago Medicine, Chicago, USA; 2 Cardiology, Center for Arrhythmia Care, University of Chicago Medicine, Chicago, USA

**Keywords:** cardiovascular implantable electronic device, cied infection, cied pocket erosion

## Abstract

Background

The CanGaroo® Envelope (Aziyo Biologics, Silver Spring, MD) is intended to securely hold a cardiovascular implantable electronic device (CIED) to create a stable environment when implanted in the body. Data on the utilization of this newly available product are limited.

Objective

In this study, our objective was to describe the specific profiles of patients who may benefit from the use of the CanGaroo® Envelope at the time of CIED implantation.

Methods

The utilization of the CanGaroo® Envelope was assessed from January 2019 to October 2019 among a series of patients who were either undergoing de-novo CIED implantation or replacement.

Results

Among a total of 50 patients, the CanGaroo® Envelope was utilized in 15 (30%). Three distinct patient profiles were identified: profile 1: elderly patients with poor tissue turgor at risk of wound dehiscence or erosion; profile 2: patients with a history of previous device infection; and profile 3: patients at high risk of device infection having one or more of the following risk factors - chronic kidney disease, immunocompromised state, or diabetes mellitus. At a mean follow-up of 18 ±3 months, no CIED pocket erosion, dehiscence, or infection was noted.

Conclusions

Three distinct profiles of patients who could potentially benefit from the use of the CanGaroo® Envelope were identified by the implanting physicians. Long-term follow-up data, including infection and wound dehiscence rates, are necessary to further analyze the optimal utilization of the device.

## Introduction

**Figure 1:**
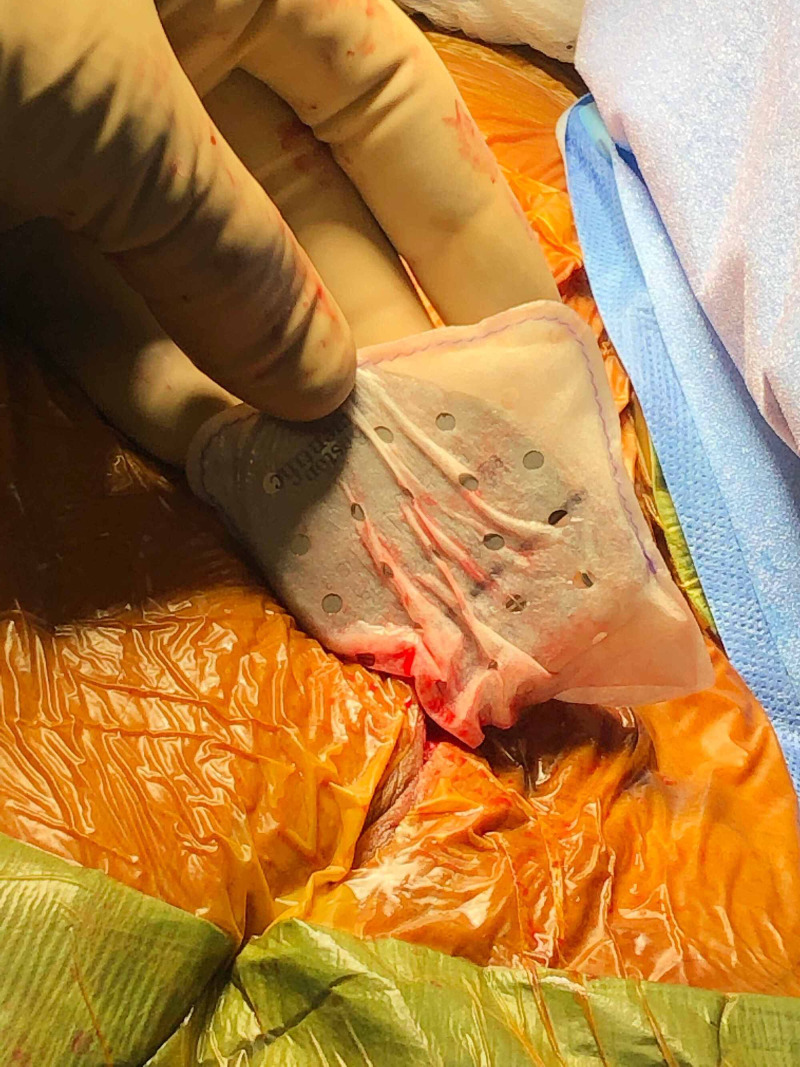
CanGaroo® Envelope conforms to the device The CanGaroo® is available in a variety of sizes (small, medium, large, extra-large, and extra-extra-large) to accommodate pacemakers and transvenous and subcutaneous defibrillators. In this example, the CanGaroo® Envelope conforms well to the permanent pacemaker generator

The CanGaroo® Envelope (Aziyo Biologics, Silver Spring, MD) is indicated to securely hold a cardiovascular implantable electronic device (CIED) to create a stable environment when implanted in the body. It conforms to the implantable device (Figure 1), supports and reinforces the pocket, and creates a stabilized environment that may reduce the risk of device erosion [1]. Data on the utilization of this newly available product are limited, particularly on the type of patients who may benefit from its use. The objective of this paper was to describe specific profiles of patients who may benefit from the use of the CanGaroo® Envelope at the time of CIED implantation or replacement.

## Materials and methods

From January 2019 to October 2019, patients who were either undergoing de-novo CIED implantation or replacement were deemed eligible to receive the CanGaroo® Envelope during their procedure. The Institutional Review Board at the University of Chicago Medicine approved the collection of these data for analysis and publication. Informed patient consent for data collection was obtained. The utilization of the CanGaroo® Envelope was left to the discretion of the implanting physician. All de-novo implantations or replacements were performed in the electrophysiology laboratory by the attending electrophysiologists by using the standard sterile technique. All patients received a pre-procedure intravenous antibiotic (cefazolin 2 grams or vancomycin 1 gram) within one hour of the incision. In all cases, the CanGaroo® Envelope was immersed for one to two minutes in a 250 cc normal saline bath infused with 80 mg gentamicin. Capsulectomy was not performed in the majority of cases and was left to the discretion of the operator. All patients underwent pocket closure using absorbable suture in three or four layers. Patients were seen in the outpatient setting in person or via telemedicine at three weeks, three months, and every 12 months thereafter for device-related interrogation (in person or remote) and visual inspection of the pocket. Endpoints included pocket erosion, dehiscence, and infection.

Categorical variables are presented as the percentage of patients, and continuous variables are presented as mean ±standard deviation for baseline characteristics and median (25th percentile, 75th percentile) for endpoints.

## Results

Patient characteristics

**Figure 2:**
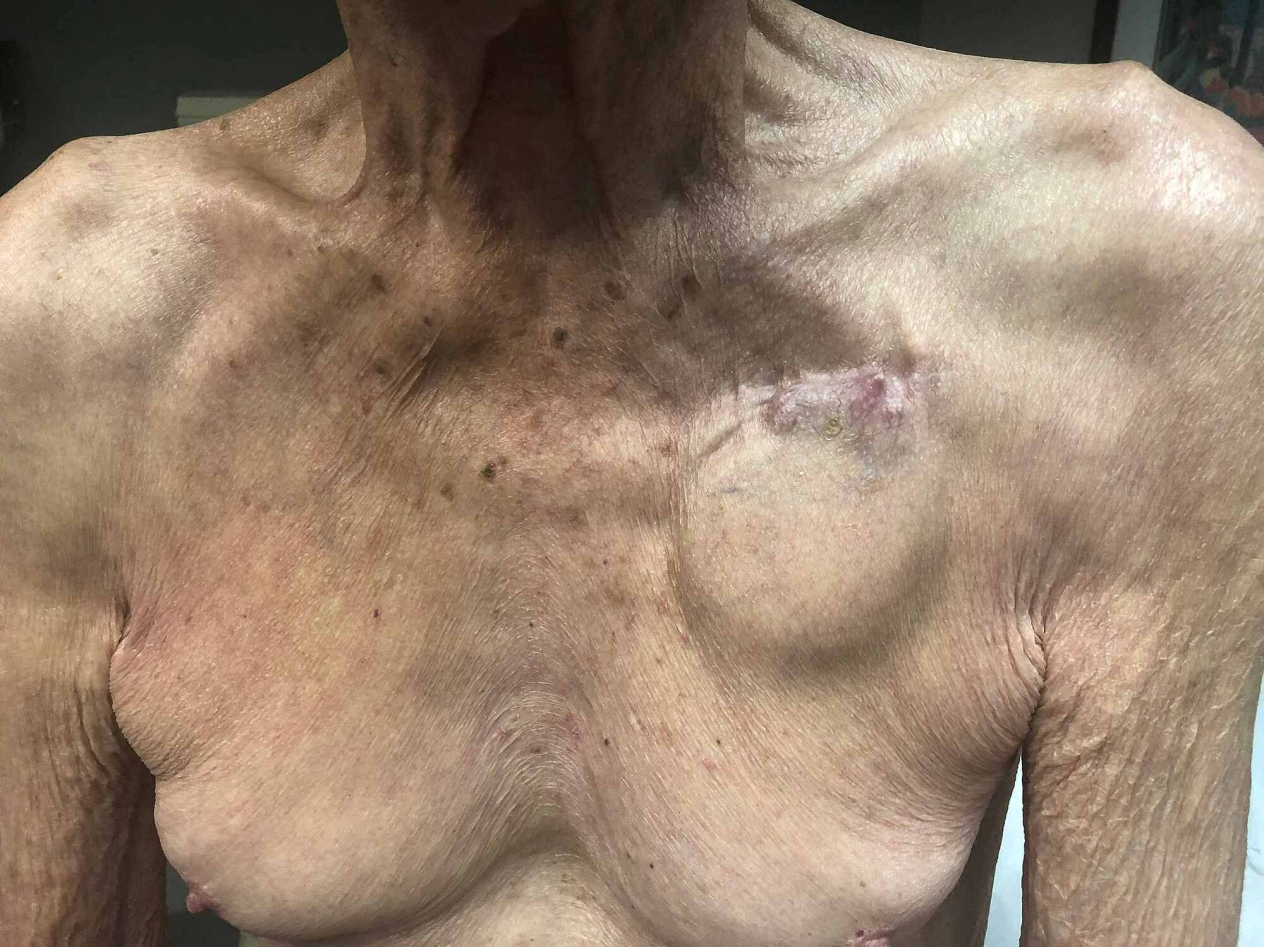
High risk for erosion A 77-year-old man with complete heart block presented for permanent pacemaker generator change secondary to elective replacement indication. He reported that the skin and soft tissue over his pacemaker had thinned over the last year, and he attributed this to a 10-pound weight loss he had suffered as a result of a chronic gastrointestinal illness. The outline of the generator was visible as were the edges of the header. The skin overlying the header was not discolored, indurated, or fixed but appeared thin. His risk of poor wound healing and erosion was high. This patient underwent a pacemaker generator change without incident and a CanGaroo® Envelope was utilized to reinforce the pocket

From January 2019 to October 2019, a total of 50 patients either underwent de-novo CIED implantation or replacement. The CanGaroo® Envelope was utilized in 15 (30%) of them. Patient characteristics are provided in Table 1. The majority of the population were men (60%). The mean age of the patients was 71 ±10 years. The prevalence of cardiovascular comorbidities such as hypertension and diabetes mellitus was high at 60% (n=9) and 80% (n=12) respectively. Three distinct patient profiles were identified: profile 1: elderly patients with poor tissue turgor at risk of wound dehiscence (Figure 2); profile 2: patients with a history of previous device infection; and profile 3: patients at high risk of device infection having one or more of the following risk factors - chronic kidney disease, immunocompromised state, or diabetes.

**Table 1:** Baseline characteristics (n=15) Continuous variables are reported as mean ±standard deviation and categorical variables are presented as percentages CIED: cardiovascular implantable electronic device; CRT-D: cardiac resynchronization therapy with defibrillator; ICD: implantable cardioverter-defibrillator; PPM: permanent pacemaker; SD: standard deviation

Variables	Values
Male gender	60%
Age (years), mean ±SD	71 ±10
Body mass index (kg/m^2^), mean ±SD	30 ±6
Race	
White	60%
African American	40%
Hypertension	60%
Diabetes mellitus	80%
Cardiac arrest	20%
Chronic obstructive pulmonary disease	13%
Ejection fraction (%), mean ±SD	40 ±12
Heart failure	60%
Coronary artery disease	10%
Atrial fibrillation	10%
End-stage renal disease	67%
Previous CIED infection	20%
Number of patients with de-novo implants (PPM)	4
Number of patients with de-novo implants (ICD or CRT-D)	6
Number of patients with replacement (PPM)	2
Number of patients with replacement (ICD or CRT-D)	3

Follow-up and endpoints

At a mean follow-up of 18 ±3 months, no CIED pocket erosion, dehiscence, or infection was noted.

## Discussion

The main findings of the present study are as follows:

1. Three distinct patient profiles were identified regarding the utilization of the CanGaroo® Envelope.

2. No CIED pocket erosion, dehiscence, or infection was encountered in the short-term follow-up in association with the use of the CanGaroo® Envelope.

CIED implantation is associated with an overall low complication rate; however, pocket issues such as dehiscence, erosion, and infection remain important causes of morbidity and mortality, and they often require total system explantation [2]. Interventions to reduce the risk of these complications are varied but they all focus on aiding wound healing. Elderly patients are particularly at high risk of poor wound healing. Sgonc and Gruber have described a number of age-related alterations in wound healing that can negatively impact or delay healing in elderly patients, and these include delayed angiogenesis, delayed collagen deposition, and delayed re-epithelialization [3].

The extracellular matrix that constitutes the CanGaroo® Envelope is derived from natural material and has distinctive properties that may aid in wound healing. In vitro and in vivo animal studies have shown that the extracellular matrix may facilitate angiogenesis [4], recruit macrophages [5], and induce reconstructive remodeling [6].

The following procedural factors have been variably associated with a lower risk of CIED infection: the administration of intravenous preoperative antibiotics, adherence to proper sterile technique, pocket washout, and the use of certain antimicrobial envelopes [7]. Patient risk factors that have been associated with a higher risk of CIED infection include a history of previous CIED infection, diabetes mellitus, end-stage renal disease (ESRD), and immunocompromised state [8].

The World-wide Randomized Antibiotic Envelope Infection Prevention Trial (WRAP-IT) study was the first large randomized study to show that the additional use of the TYRX™ antimicrobial envelope was associated with a lower risk of CIED infection [9]. Unlike the CanGaroo® Envelope, it is a synthetic product. It is important to mention that the CanGaroo® Envelope has not been studied in a randomized trial to reduce CIED infection, even though in vitro and in vivo animal studies have shown a reduction in staphylococcus colony growth and infection associated with its use [10].

There are a number of limitations to this analysis. The utilization of the CanGaroo® Envelope was left to the discretion of the implanting physician; patients were not randomized. The overall patient population was small and the follow-up period was short. Endpoints evaluated such as erosion and infection are more commonly encountered years after pocket intervention.

## Conclusions

Three patient profiles were identified regarding the utilization of the CanGaroo® Envelope in patients undergoing de-novo implantation or replacement of CIEDs. Long-term follow-up data, including infection and wound dehiscence rates, are necessary to further analyze the optimal utilization of the device.
